# Dual-Structure PVDF/SDS Nanofibrous Membranes for Highly Efficient Personal Protection in Mines

**DOI:** 10.3390/membranes12050482

**Published:** 2022-04-29

**Authors:** Gang Zhou, Rulin Liu, Qingfeng Xu, Kaili Wang, Yongmei Wang, Seeram Ramakrishna

**Affiliations:** 1 College of Safety and Environmental Engineering, Shandong University of Science and Technology, Qingdao 266590, China; zhougang@sdust.edu.cn (G.Z.); xqf_sdust@163.com (Q.X.); 17854337811@163.com (K.W.); yongmeiwang2011@163.com (Y.W.); 2 State Key Laboratory of Mining Disaster Prevention and Control Co-Founded by Shandong Province and the Ministry of Science and Technology, Shandong University of Science and Technology, Qingdao 266590, China; 3 Department of Mechanical Engineering, National University of Singapore, Singapore 117574, Singapore; seeram@nus.edu.sg

**Keywords:** electrospinning method, individual protection equipment, polyvinylidene fluoride, co-blending modification, nanofiber membrane

## Abstract

Pneumoconiosis in miners is considered a global problem. Improving the performance of individual protective materials can effectively reduce the incidence of pneumoconiosis. In this study, the blend membrane of sodium dodecyl sulfate and polyvinylidene fluoride with a dual structure was prepared using electrospinning techniques, and the morphological structure, fiber diameter, and filtration performance of the nanofiber membranes were optimized by adjusting the PVDF concentration and SDS content. The results show that the incorporation of SDS enabled the nanofiber membranes to show tree-like and beaded fibers. Compared with the original PVDF membrane, the small content of tree-like fibers and beaded fibers can improve the filtration efficiency and reduce the resistance of the fiber membrane. The prepared nanofiber membrane has excellent comprehensive filtration performance, and the quality factor is 0.042 pa^−^^1^ when the concentration of PVDF is 10 wt% and the addition of SDS is 0.1 wt%. Furthermore, after high-temperature treatment, the membrane could still maintain good filtration performance. The PVDF/SDS blend nanofiber membrane has outstanding filtration efficiency and good thermal stability and can fully meet the personal protection of miners in underground high-temperature operation environments.

## 1. Introduction

Pneumoconiosis is currently one of the most serious occupational diseases in China, and it is mainly found in the mining industry. The concentration of respirable dust directly affects the incidence of pneumoconiosis [1]. In order to solve the problem of high dust concentration in coal mines, the current methods of dust reduction using ventilation, dust removal equipment, and coal seam water injection [2,3,4,5,6,7,8,9,10] have played a positive and effective role in improving the working environment and reducing the dust concentration at the working face. However, the above measures are mostly physical–chemical methods, and the concentration of respiratory dust is still very high. It can be seen that it is of great practical significance to carry out research on individual protection equipment for coal miners and effectively make the last line of protection for coal miners.

Individual protection equipment mainly applies fiber filter membranes to achieve efficient filtration of dust particles, and the performance of the filter membrane directly affects whether it can play an effective protective role for miners. Polyvinylidene fluoride has excellent mechanical properties [11,12,13,14], chemical resistance, biocompatibility, etc. These excellent properties of PVDF and the structural uniqueness of electrospun nanofibers make PVDF nanofiber membranes widely used [15,16,17,18]. However, because the fiber diameter of PVDF fiber membranes is still larger, the filtration performance is difficult to improve further. Various strategies have been proposed to improve PVDF membrane properties and filtration performance. Li et al. [19] designed PVDF tree-like nanofiber webs and investigated the filtration properties of the membranes with different basis weights. Li et al. [20] fabricated a PVDF nanofiber/nanonet air filter by adding anionic surfactant. This membrane could be a promising candidate in various air filtration applications. Liu et al. [21] developed a novel electrospun nanofiber film PVDF–ESNF to maintain the removal efficiency of ultrafine particulates at more than 86.9 wt%. Most studies were conducted at a flow rate of 32 L/min to remove airborne particulate matter (PM). However, there is a special environment of high dust concentration, high temperature, and high labor intensity in mines [10]. The research on membranes must be carried out at a high flow rate and achieve the best filtration performance. To further develop mine personal protective equipment that is comfortable, low respiratory resistance, high filtration efficiency, and good thermal stability are of great significance.

Therefore, this study aimed to prepare a dual-structure individual protective filter membrane for miners using a co-blending modification of PVDF. Furthermore, in order to further obtain more perfect fiber membrane materials, the effects of the polymer concentration as well as the additive content on the morphology and filtration performance of the nanofibers were investigated. Thus, the application of electrospun PVDF nanofibers for dust masks in coal mines is further expanded.

## 2. Methods

### 2.1. Materials

Polyvinylidene fluoride (PVDF, M_W_ = 534,000), N,N-Dimethylformamide (DMF, 99.8%), acetone (ACS reagent, ≥99.5%), and sodium dodecyl sulfate (SDS, ≥98.5%) were purchased from Sigma-Aldrich. All chemicals were used without further purification in this work.

### 2.2. Experimental Process

#### 2.2.1. Preparation of Spinning Solution

At room temperature, PVDF and SDS were dissolved in the mixed solvent comprised of acetone and DMF at a ratio of 1:1 and stirred with a magnetic stirrer for more than 12 sh until a uniform spinning solution was formed. The concentration of PVDF in the solution were 8%, 10%, and 12 wt%, and the concentrations of SDS were 0.1, 0.2, and 0.3 wt%, respectively.

#### 2.2.2. Preparation of Nanofiber Membrane

**Figure 1:**
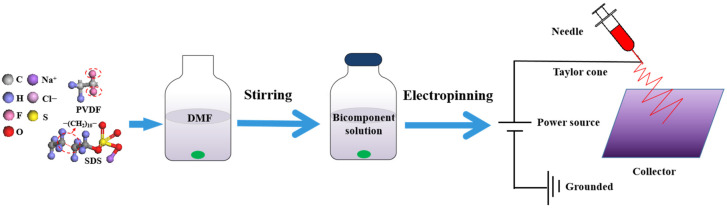
Schematic representation of experimental process.

The spinning solution was injected into the syringe with a metal needle, and the inner diameter of the needle was 0.7 mm × 38 mm. Then, the syringe was fixed on the syringe pump and connected to the spinneret through a tube. The feed rate was 0.8 mL/h, and the spinneret speed was 10 mm/s. A flat plate was placed under the spinneret. The distance between the needle and the flat plate was 10 cm. The positive pole of the high-voltage power supply was connected to the spinneret, and the negative pole was connected to the flat plate. The voltage was 20 kV. In a high-voltage electric field, because of the action of the electric field force, the spinning solution overcomes the surface tension and ejects from the top of the Taylor cone to form a charged jet. The jet is further stretched, accompanied by solvent volatilization and solidification, and finally forms a nanofiber membrane to be deposited on the flat plate. The schematic representation of the experimental process is shown in Figure 1.

### 2.3. Characterization

The morphology and structure of the prepared nanofiber films were studied using a scanning electron microscope, and the diameter of the fibers was measured and calculated using Image-Pro Plus. XRD and FTIR were used to analyze the chemical structure properties of nanofiber membranes. The thermal stability of the nanofiber membrane was tested using a thermogravimetric analyzer. The test process involved raising the temperature from 30 °C to about 600 °C under N_2_ conditions. The membrane was coated with supporting non-woven fabric to test the filtration performance. The filtration performance (filtration efficiency and pressure drop) of the membrane was obtained using a tester conforming to the Chinese mask standard GB2626. The flow rate was set to 85 L/min, and the test area was 100 cm^2^. The aerosol used in the test was composed of NaCl particles with a diameter of 0.3 um. Each sample was tested three times, and the average value was taken. The quality factor [22] was used to evaluate the comprehensive filtration performance of membranes, and the calculation formula is as follows:

$$QF=-\frac{\ln\left(1-\eta \right)}{\Delta p}$$

where *QF* denotes the quality factor; *η* denotes the filtration efficiency; Δ*p* denotes the pressure drop.

## 3. Results and Discussion

### 3.1. Morphological Analysis of Nanofiber Membranes

**Figure 2:**
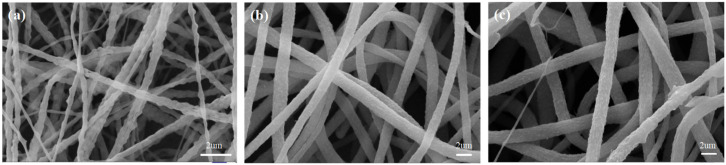
SEM images of PVDF nanofiber membranes with different concentrations: (**a**) 8 wt%, (**b**) 10 wt%, and (**c**) 12 wt%.

As shown in Figure 2, at 8 wt% PVDF concentration, the fibers appeared to be bonded, and the average fiber diameter was about 0.4 um as measured by Image-pro Plus. As the polymer concentration increased, the fiber diameter gradually increased, and the distribution uniformity was also relatively improved. At 10 wt% PVDF, the average diameter was about 1.1 um, and at 12 wt% PVDF, the average diameter was about 1.6 um.

**Figure 3:**
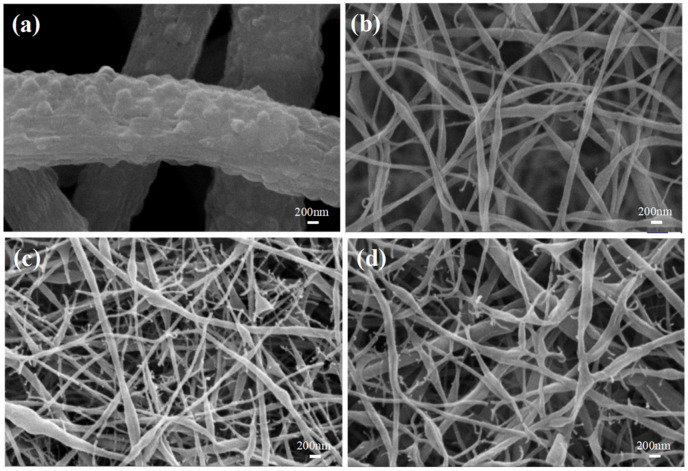
SEM images of PVDF/SDS nanofiber membranes with different SDS concentrations: (**a**) 0% wt, (**b**) 0.1 wt%, (**c**) 0.2 wt%, and (**d**) 0.3 wt%.

**Figure 4:**
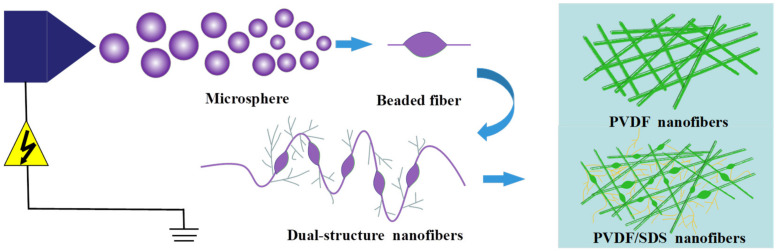
Forming mechanism of PVDF/SDS nanofibers.

As shown in Figure 3, compared with the PVDF original membrane, the diameter of the PVDF/SDS modified membrane was significantly smaller. In addition, PVDF/SDS nanofibers showed tree-like [23] and beaded structures [24]. The forming mechanism is shown in Figure 4. The formation of tree-like nanofibers comprised of trunk fibers and branch fibers was observed and corresponded to the increased conductivity caused by the incorporation of SDS. In addition, the electric forces overcame the surface tension. This led to a greater stretching force on the jet, which facilitated the splitting of the jet in the electric field and made it easier to obtain finer fibers. With increasing SDS content, the conductivity of the spinning solution increased, and the surface tension decreased [20,23]. Thus, the formation and stabilization of the Taylor cone were affected, and the stretching of the jet was hindered, which promoted the aggregation of some of the solution to form beads. The intermolecular interaction introduced between the PVDF molecules and the surfactant SDS may have adjusted the molecular structure of the polymer, thus changing the solution properties, and may have also promoted the generation of this morphology.

When the content of SDS was 0.1 wt%, fewer tree-like nanofibers were observed, which may be due to the low electrical conductivity of the solution. The tree-like nanofibers were composed of trunk fibers with a 113 nm average diameter and branch fibers with a 38 nm average diameter. The average diameter of beaded fibers was 202 nm. As the content of SDS increased to 0.2%, the uniformity and content of tree-like branch fibers increased. The tree-like nanofibers were composed of trunk fibers with a 63 nm average diameter and branch fibers with a 36 nm average diameter. The average diameter of the beaded fibers was 178 nm. This was because the incorporation of SDS significantly increased the electrical conductivity of the solution and the charge density of the jet, which caused the jet to split and form fine fibers.

**Figure 5:**
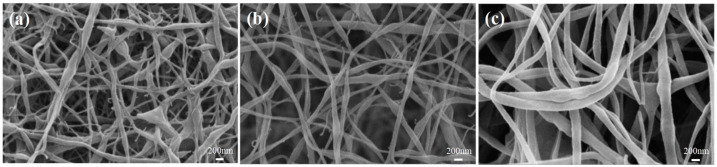
SEM images of PVDF/SDS nanofiber membrane with different PVDF concentrations: (**a**) 8 wt%, (**b**) 10 wt%, and (**c**) 12 wt%.

As the content of SDS increased to 0.3 wt%, the electrical conductivity of the solution became too large, and the spinning process was unstable. The tree-like nanofibers were composed of trunk fibers with a 105 nm average diameter and branch fibers with a 37 nm average diameter. The average diameter of the beaded fibers was 207 nm. When the concentration of SDS was constant, SEM images of the PVDF/SDS nanofiber membrane were taken, as shown in Figure 5.

As shown in Figure 4, with an increasing concentration of PVDF, the number of tree-like fibers gradually reduced, and the fiber diameter increased with a constant SDS concentration. It can be seen that although SDS increased the conductivity of the solution, the conductivity decreased as the concentration of PVDF increased, and the high viscosity of the solution inhibited the draw refinement of the fibers, which caused the fiber diameter to become thicker. These results indicate that the concentration of PVDF and SDS can strongly affect the structure and morphology of nanofibers.

### 3.2. The Chemical Structure Properties of Nanofiber Membranes

**Figure 6:**
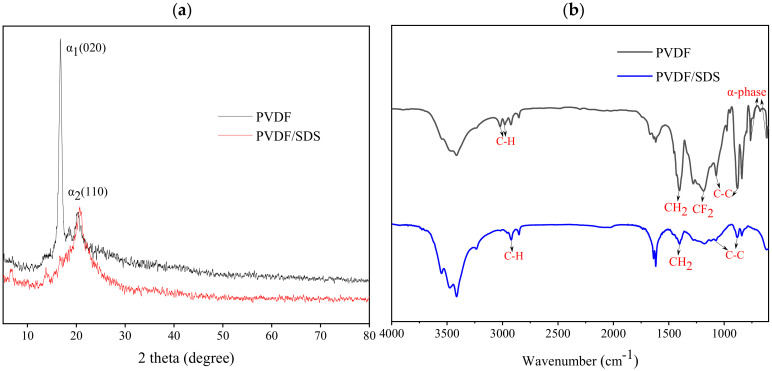
(**a**) XRD patterns of nanofiber membranes, (**b**) FTIR spectra of nanofiber membranes.

From the XRD spectra in Figure 6a, it can be seen that the PVDF fiber membrane showed a weak peak at 2θ = 16.82° and a strong peak at 2θ = 20.30°, which corresponded to the α-crystalline phase with the reflection planes of (020) and (110). After SDS blending modification, all the above diffraction peaks vanished, and the PVDF/SDS nanofiber membrane had no crystal structure. The reason might be that SDS molecules interact with PVDF molecules and finally present an amorphous state. As can be seen from the FTIR spectra in Figure 6b, the typical characteristics of the α-phase of the PVDF fiber membrane were about 610 cm^−1^ and 760 cm^−1^. The peaks at 3020 cm^−1^ and 2980 cm^−1^ were the stretching vibrations of C-H, and the peak at 1402 cm^−1^ was the deformation and rocking vibration of CH_2_. The peak at 1180 cm^−1^ was the stretching vibration of CF_2_. The peaks at 1180 cm^−1^ and 880 cm^−1^ were assigned to the vibration of C-C. However, the characteristic absorption peaks of CF_2_ and the α-phase did not appear in the PVDF/SDS nanofiber membrane. These results were in accordance with the XRD analysis. Thus, it can be speculated that the interaction between the low surface energy of CF_2_ and the SDS molecules contributes to the formation of the nanofiber membrane structure.

### 3.3. The Analysis of Thermal Stability

**Figure 7:**
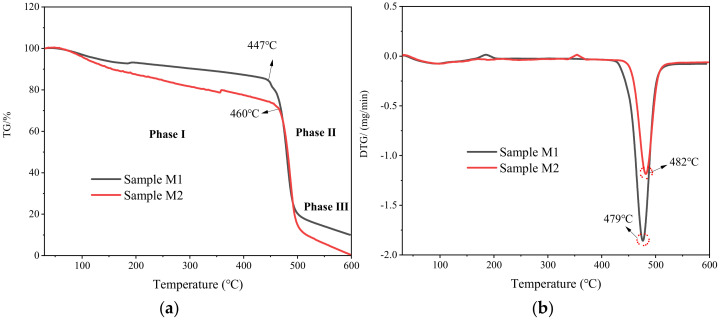
TG and DTG curves of (**a**) PVDF nanofiber membrane-M1 and (**b**) PVDF/SDS nanofiber membrane-M2.

The thermal stability of the PVDF/SDS membrane (sample M2) was analyzed and compared with the original PVDF membrane (sample M1). Figure 7 shows the TG and DTG curves of M1 and M2 at 30–600 °C. The pyrolysis process of the filter membrane included three stages. The first stage was mainly the volatilization of the solvent and the preheating process of the sample. The TG curve decreased slightly, and the DTG curve reflected that the thermal weight loss rate remained unchanged. The second stage was mainly the decomposition process of the PVDF main chain, and the TG and DTG curves decreased sharply. The decomposition temperature of the PVDF filter membrane was about 427 °C, and the decomposition temperature of the PVDF/SDS filter membrane was about 441 °C. The third stage was the slow decomposition process of residues, which is mainly the process of continuous heat loss of substances that are difficult to decompose by pyrolysis. The temperatures corresponding to the maximum weight loss rate of the M1 and M2 filter membranes are 479 °C and 482 °C, respectively. The results showed that the thermal decomposition temperature of the modified PVDF fiber membrane (sample M2) is increased and has better thermal stability.

### 3.4. The Filtration Performance of Nanofiber Membranes

**Figure 8:**
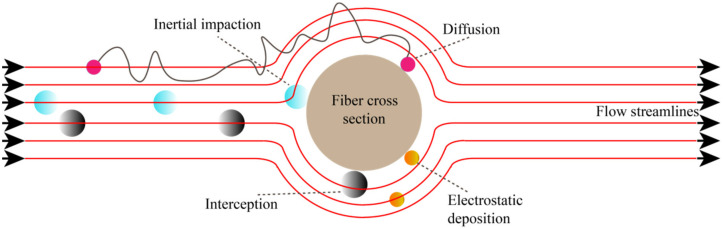
Filtration mechanism of membranes [10].

Fiber membrane filtration [25,26] occurs mainly through the action of force (inertial force, electrostatic force, and van der Waals force) and the internal structure of materials (fiber diameter and fiber bulk density). Generally, when the filter membrane filters dust particles, several effects work at the same time. Figure 8 shows the schematic diagram of the filtration mechanism.

To obtain nanofiber membranes with better filtration performance, the effects of the PVDF and SDS content on the filtration efficiency, pressure drop, and quality factor of the fiber membranes were studied. Figure 6 shows the filtration efficiency and resistance of the original PVDF and PVDF/SDS (0.1 wt% SDS).

**Figure 9:**
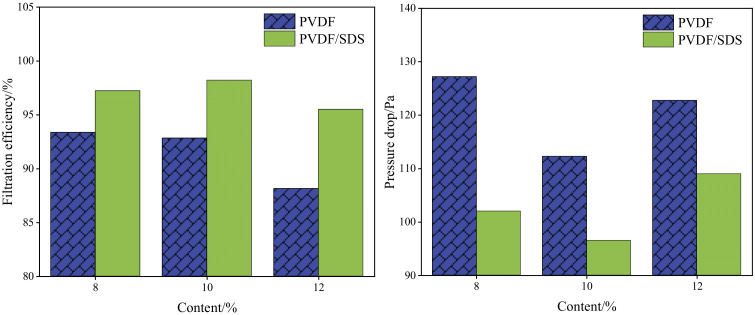
Filtration efficiency and pressure drop of membranes.

**Figure 10:**
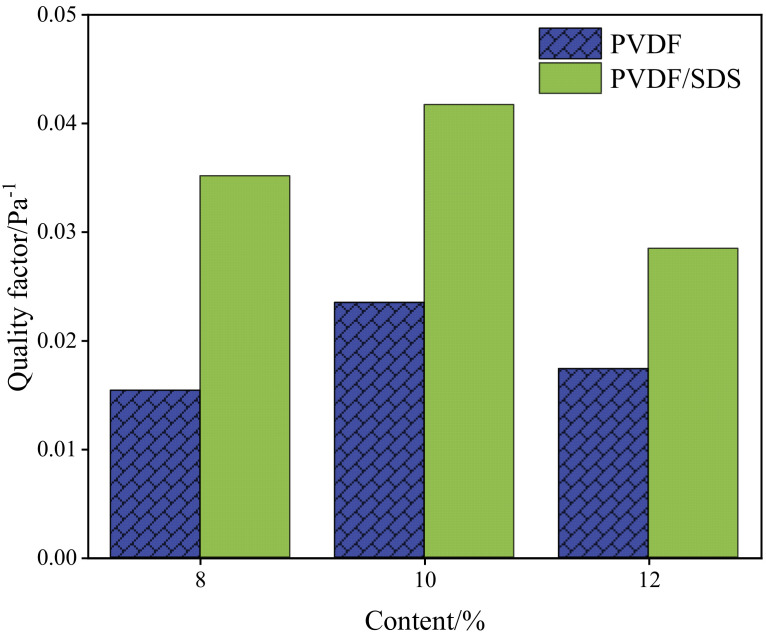
Quality factor of membranes.

As shown in Figure 9, the PVDF/SDS nanofiber has higher filtration efficiency and lower filtration resistance than the original PVDF membrane. That is because the tree-like nanofibers are composed of trunk fibers and branch fibers. The overlapping of coarse and fine fibers can reduce the bulk density, increase air permeability, and reduce filtration resistance. At the same time, the branch fibers can capture finer particles, and thus the filtration efficiency improves. The filtration performance of the fiber membrane was comprehensively evaluated by the quality factor (Figure 10).

**Figure 11:**
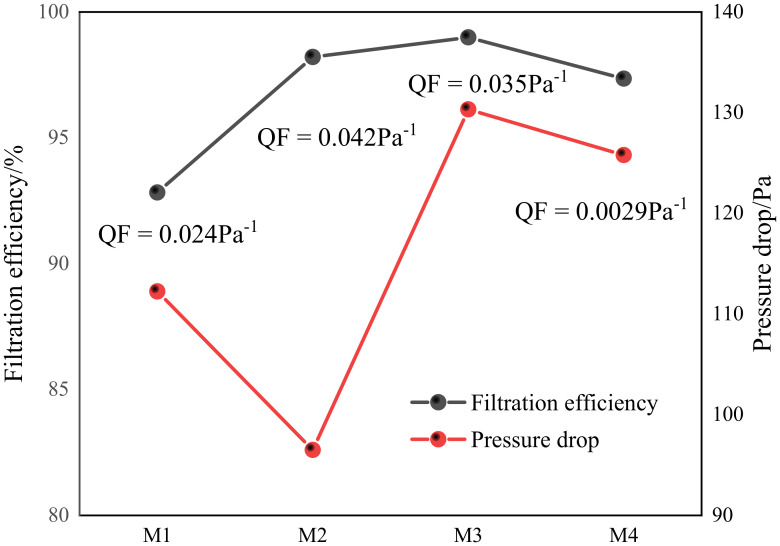
Filtration performance of PVDF/SDS nanofiber membranes with different SDS concentrations.

As shown in Figure 10, when the content of PVDF was 10 wt%, the quality factor was highest. To further study the impact of SDS content on filtration performance, Figure 11 shows that the content of PVDF is 10 wt%, the content of SDS is 0 wt%, 0.1 wt%, 0.2 wt%, 0.3 wt%, which are recorded as M1, M2, M3, and M4.

As shown in Figure 11, the filtration performance was significantly improved by adding SDS. However, with the increase in SDS content to 0.2 wt%, there were more branch fibers, and the pressure drop increased sharply. When the SDS concentration increased to 0.3 wt%, the jet began to be unstable. The fiber diameter increased, according to the SEM images, resulting in a reduction in the pressure drop. In conclusion, when the PVDF content is 10 wt% and SDS content is 0.1 wt%, the PVDF/SDS fiber membrane has the best filtration performance. The quality factor is the highest, which is 0.042 pa^−1^. After heat treatment, the membrane retained a low pressure drop (96 ± 5 Pa) and high filtration efficiency (98.2 ± 0.2%).

## 4. Conclusions

This work developed a new promising dual-structure PVDF/SDS nanofiber membrane. The surface morphology, fiber diameter, chemical structure, filtration performance, and thermal stability of the prepared membranes were characterized by SEM, XRD, FTIR, automated filter tester, and TG-DTG. The PVDF/SDS nanofiber membrane shows low pressure drop, high filtration efficiency, good thermal stability, and high temperature resistance, and it has potential in the rapidly developing field of respiratory protection equipment in mines. However, the use of organic solvents may affect environmental safety and human health. In the future, green solvent and solvent-free synthesis technology will be further studied to realize the goal of healthy and sustainable development.

## Data Availability

Data is contained within the article.

## References

[1] Shekarian Y., Rahimi E., Shekarian N., Rezaee M., Roghanchi P. (2021). An analysis of contributing mining factors in coal workers’ pneumoconiosis prevalence in the United States coal mines, 1986–2018. Int. J. Coal Sci. Technol..

[2] Liu M.P., Lin M.Y., Hu S.P., You X.F., Li L. (2021). Effect of biomass surfactant on dehydration performance of low-rank coal ant its mechanism. J. Shandong Univ. Sci. Technol. (Nat. Sci.).

[3] Han W.B., Zhou G., Wang J.P., Jiang W.J., Zhang Q., Kong Y., Miao Y.N. (2021). Experimental investigation on combined modifcation for micro physicochemical characteristics of coal by compound reagents and liquid nitrogen freeze-thaw cycle. Fuel.

[4] Zhang K.X., Zhang J., Wei J.P., Ren T., Xu X.Y. (2019). Coal seam water infusion for dust control: A technical review. Environ. Sci. Pollut. Res..

[5] Zhang Q.T., Zhou G., Hu Y.Y., Xing M.Y., Zhang R., Wang P.F., Hu S.Y. (2021). Microwetting dynamic behavior and mechanism for coal dust based on low feld NMR method—A case study. Fuel.

[6] Li S.L., Zhou G., Liu Z.Q., Wang N.G., Wei Z.Y., Liu W. (2020). Synthesis and performance characteristics of a new ecofriendly crust-dust suppressant extracted from waste paper for surface mines. J. Clean. Prod..

[7] Zhou G., Zhang Q.T., Hu Y.Y., Gao D.H., Wang S.C., Sun B. (2020). Dust removal effect of negatively-pressured spraying collector for advancing support in fully mechanized coal mining face: Numerical simulation and engineering application. Tunn. Undergr. Space Technol..

[8] Ding J.F., Zhou G., Liu D., Jiang W.J., Wei Z.Y., Dong X.S. (2020). Synthesis and performance of a novel high-efficiency coal dust suppressant based on self-healing gel. Environ. Sci. Technol..

[9] Jiang W.J., Zhou G., Duan J.J., Liu D., Zhang Q.T., Tian F.C. (2021). Synthesis and characterization of a multifunctional sustained release organic−inorganic hybrid microcapsule with self-healing and flame-retardancy properties. Appl. Mater. Interfaces.

[10] Liu R.L., Ji X.D., Zhou G., Liu Z.Q., Xu Q.F., Ramakrishna S. (2021). Electrospun nanofibers for personal protection in mines. Chem. Eng. J..

[11] Sun Q.Q., Leung W.W.F. (2019). Charged PVDF multi-layer filters with enhanced filtration performance for filtering nano-aerosols. Sep. Purif. Technol..

[12] Song Y.S., Yun Y.H., Lee D.Y., Kim B.Y. (2021). Effect of PVDF Concentration and Number of Fiber Lines on Piezoelectric Properties of Polymeric PVDF Biosensors. Fibers Polym..

[13] Spasova M., Manolova N., Markova N., Rashkov I. (2016). Superhydrophobic PVDF and PVDF-HFP nanofibrous mats with antibacterial and anti-biofouling properties. Appl. Surf. Sci..

[14] Li X.X., Ji D.X., Yu B., Ghosh R., He J.H., Qin X.H., Ramakrishna S. (2021). Boosting piezoelectric and triboelectric effects of PVDF nanofiber through carbon-coated piezoelectric nanoparticles for highly sensitive wearable sensors. Chem. Eng. J..

[15] Li M., Sun J.X., Chen G., Yao S.Y., Cong B.W. (2022). Construction double electric field of sulphur vacancies as medium ZnS/Bi2S3-PVDF self-supported recoverable piezoelectric film photocatalyst for enhanced photocatalytic performance. Appl. Catal. B Environ..

[16] van Goethem C., de Beeck D.O., Ilyas A., Thijs M., Koeckelberghs G., Aerts P.E.M., Vankelecom I.F.J. (2021). Ultra-thin and highly porous PVDF-filters prepared via phase inversion for potential medical (COVID-19) and industrial use. J. Membr. Sci..

[17] Xiong J.P., Shao W.L., Wang L., Cui C., Gao Y.F., Jin Y.R., Yu H.Q., Han P.J., He J.X. (2022). High-performance anti-haze window screen based on multiscale structured polyvinylidene fluoride nanofibers. J. Colloid Interface Sci..

[18] Shen X.L., Deng Z.J.N.P., Fan J., Wang L., Xia Z.P., Liu Y., Liu J. (2021). Rational designing of tree-like polymer gel membrane based on PVDF/lamellar organic montmorillonite nanofiber with excellent flame retardancy and superior ion conductivity for high-performance lithium-ion capacitor. Chem. Eng. J..

[19] Li Z.J., Kang W.M., Zhao H.H., Hu M., Ju J.G., Denga N.P., Cheng B.W. (2016). Fabrication of a polyvinylidene fluoride tree-like nanofiber web for ultra high performance air filtration. Rsc Adv..

[20] Li X., Wang C., Huang X.H., Zhang T.H., Wang X.F., Min M.H., Wang L.M., Huang H.L., Hsiao B.S. (2018). Anionic Surfactant-Triggered Steiner Geometrical Poly(vinylidene fluoride) Nanofiber/Nanonet Air Filter for Efficient Particulate Matter Removal. ACS Appl. Mater. Interfaces.

[21] Liu G., Nie J., Han C., Jiang T., Yang Z., Pang Y., Xu L., Guo T., Bu T., Zhang C. (2018). Self-Powered Electrostatic Adsorption Face Mask Based on a Triboelectric Nanogenerator. ACS Appl. Mater. Interfaces.

[22] Mei Y., Wang Z.M., Li X.S. (2013). Improving Filtration Performance of Electrospun Nanofiber Mats by a Bimodal Method. J. Aoolied Polym. Sci..

[23] Li Z.J., Xu Y.Z., Fan L.L., Kang W.M., Cheng B.W. (2016). Fabrication of polyvinylidene fluoride tree-like nanofiber via one-step electrospinning. Mater. Des..

[24] Zhao X.L., Li Y.Y., Hua T., Jiang P., Yin X., Yu J.Y., Ding B. (2017). Low-resistance dual-purpose air filter releasing negative ions and effectively capturing PM2.5. ACS Appl. Mater. Interfaces.

[25] Fu Q.F., Fang J., Shi J., Cao X.Q., Lv X.J. (2019). Effect of particulate matter on dust removal performance of polyacrylonitrile fiber bundle filter. J. Shandong Univ. Sci. Technol. (Nat. Sci.).

[26] Liu R.L., Zhou G., Wang C.M., Jiang W.J., Wei X. (2020). Preparation and performance characteristics of an environmentally-friendly agglomerant to improve the dry dust removal effect for filter material. J. Hazard. Mater..

